# Guselkumab in Psoriatic Arthritis: Therapeutic Impact on Axial and Peripheral Involvement—Monocentric Real-World Evidence

**DOI:** 10.3390/jcm14093151

**Published:** 2025-05-01

**Authors:** Roberta Foti, Giorgio Amato, Elisa Visalli, Ylenia Dal Bosco, Francesco De Lucia, Angelo Montana, Giambattista Privitera, Placido Romeo, Fabio Aiello, Maria Gabriella Paolì, Rosario Foti

**Affiliations:** 1 Division of Rheumatology, AOU Policlinico “G. Rodolico” San Marco, 95121 Catania, Italyyleniadalbosco@gmail.com (Y.D.B.); rosfoti5@gmail.com (R.F.); 2 University of Enna “Kore”, 94100 Enna, Italy; fabio.aiello@unikore.it; 3 Division of Radiology San Marco Hospital, AOU Policlinico “G. Rodolico” San Marco, 95121 Catania, Italy

**Keywords:** psoriatic arthritis (PsA), spondyloarthritis (SpA), axial psoriatic arthritis (axPsA), ankylosing spondylitis (AS), Guselkumab, MRI, SPARCC, real-world evidence, interleukin 23 (IL-23), biologic drugs

## Abstract

**Background:** Axial involvement in psoriatic arthritis (axPsA) presents clinical and radiological differences from ankylosing spondylitis (AS), which may influence the therapeutic response. While Guselkumab has demonstrated efficacy in peripheral PsA, its role in axPsA is less well established, particularly in real-world settings. **Objective:** To evaluate the positive effects of Guselkumab therapy in patients with psoriatic arthritis (PsA), 58.6% of whom have axial involvement, in a 12-month, single-center, longitudinal, prospective observational cohort study conducted in a real-life setting. **Methods:** A cohort of 99 patients with PsA, including 58 with axial involvement (axPsA), was treated with Guselkumab for 12 months. Treatment efficacy was assessed by evaluating the reduction in mBASDAI, ASDAS, DAPSA, VAS Pain, LEI, and HAQ scores. The Friedman test was used to analyze whether the overall changes from baseline to 12 months were statistically significant. Patients with axial involvement were assessed by MRI, with scores measured at baseline (t_0_), after 6 months (t_6_), and after 12 months (t_12_) of therapy. Statistical evaluation was conducted using the Friedman test, followed by pairwise comparisons of values obtained at different follow-up time points using the Wilcoxon signed-rank test. Additionally, the drug’s retention rate was examined using a Kaplan–Meier curve. **Results:** After 12 months of therapy, a statistically significant reduction was observed in all clinimetric parameters. Patients with axial involvement were also evaluated by MRI at baseline, after 6 months, and after 12 months of therapy. MRI images showed a reduction in bone marrow edema and a decrease in signal intensity, indicating a significant reduction in inflammation and confirming the drug’s efficacy. Retention rate values demonstrate that Guselkumab is well tolerated and effective in the long term for the majority of patients. **Conclusions:** This 12-month real-world study of 99 PsA patients confirms the efficacy of Guselkumab in reducing disease activity in both peripheral and axial forms. The findings align with previous RWE and clinical trials (DISCOVER-1 and -2), supporting its clinical utility in PsA and axPsA, with high treatment retention.

## 1. Introduction

### 1.1. Axial Involvement in Psoriatic Arthritis (PsA) and Ankylosing Spondylitis (AS)

Psoriatic arthritis (PsA) is a chronic inflammatory disease whose main musculoskeletal manifestation is arthritis affecting one or more joints [1,2,3]. In 1973, Moll and Wright classified PsA into five subtypes—distal, polyarticular, oligoarticular, axial, and mutilating arthritis. This classification is still valid today and highlights the heterogeneity of the disease [4]. Currently, there is no clear and universally accepted definition of axial psoriatic arthritis (axPsA), as this subtype shares many features with ankylosing spondylitis (AS), another form of chronic inflammatory arthritis [5,6]. A hallmark of PsA is enthesitis, which is inflammation of the connective tissue between bone and tendon (enthesis) [7]. Interleukin-23 (IL-23), a cytokine involved in both innate and adaptive immunity that activates T helper (Th17) lymphocytes, stimulating the production of interleukin 17 (IL-17), is strongly implicated in localized inflammation at tendon–bone insertion sites, playing a key role in enthesitis [8,9]. A PsA patient’s synovium shows a high expression of IL-17A and IL-17 receptors, a condition that leads to osteochondral destruction via the proliferation of osteoclast precursors [10]. At the same time, bone erosion is followed by new bone formation. IL-22, also produced by Th17 cells, is closely involved in bone formation; it promotes proliferation of human mesenchymal stem cells and induces their differentiation into osteoblasts, also promoting enthesis and periosteal ossification, as it is a signal transducer and activator of transcription-3 (STAT3) in osteoblasts [11].

To date, several studies have demonstrated the role of TNF and the IL-23/Th17 axis in disease development, highlighting the presence of Th cell dysregulation at the core of PsA pathogenesis [12]. In all SpA (spondyloarthritis) conditions, there is a relationship between inflammation, erosion, and new bone formation. When the involvement is axial, such as in the spine, both destruction and new bone growth (syndesmophytes) occur.

However, in most cases of PsA with axial involvement, enthesitis primarily affects soft ligamentous tissues in the form of “ligamentitis”, with lesions characterized by syndesmophytosis centered on soft tissues, including ossification of the paravertebral soft tissues and sacroiliac soft ligaments, rather than fusion of joint cavities. The bony enthesis and soft tissues have radically different compositions of immune and stromal cells, which contribute to differing responses to immunomodulatory therapies, particularly IL-23 inhibition [13,14]. Precisely because axPsA and AS share certain features, including axial involvement and chronic inflammation, it is important to highlight their clinical, radiographic, and pathogenic differences in order to emphasize the need to treat them as distinct entities [15].

### 1.2. Differences in Clinical Features

AxPsA occurs in 25–70% of patients with long-standing PsA, but in only 5–28% of new cases, with onset typically around the age of 50 in both sexes [16]. In contrast, axial spondyloarthritis (axSpA) more frequently affects young males (20–30 years). Compared to axSpA, axPsA is associated with older age, higher BMI, greater peripheral involvement, the presence of psoriasis, and a lower frequency of HLA-B27 (30% vs. 85%). Other alleles, such as HLA-C06:02 and HLA-B39:01, are implicated in PsA. AxPsA more commonly presents with enthesitis, dactylitis, and asymmetric arthritis, whereas inflammatory back pain is less frequent (45%). Given the high prevalence of mechanical back pain, it is essential to distinguish inflammatory pain through a detailed medical history [17].

AxPsA negatively impacts quality of life and worsens the clinical course compared to peripheral PsA.

### 1.3. Imaging and Diagnostic Differences

Radiographic and MRI images show marked differences between axPsA and AS. In axPsA, syndesmophytes tend to be asymmetric, large, and nonmarginal, while sacroiliitis is typically less severe and often asymmetric. Periostitis, which is related to peripheral inflammation, is common. In contrast, in AS, syndesmophytes are symmetric and marginal, with the classic “bamboo spine” appearance, and there is greater spinal stiffness with involvement of the apophyseal joints [18,19,20]. Fewer than half of patients with radiographic axPsA present with evident symptoms; therefore, MRI is essential for early diagnosis.

MRI images also reveal differences in lesion symmetry and distribution, highlighting changes such as bone marrow and soft tissue edema, inflammation of the anterior longitudinal ligament, paravertebral muscles, discitis, and involvement of vertebrae and entheses [21,22,23].

### 1.4. Pathogenetic Differences

Pro-inflammatory mediators, particularly IL-23, stimulate the proliferation of Th17 lymphocytes and produce inflammatory cytokines, such as IL-6, IL-17, and IL-22, which induce inflammation at the entheses [24,25]. Alterations in the gut microbiota (dysbiosis) may contribute to the activation of inflammatory pathways, creating a gut–bone marrow–enthesis axis that plays a critical role in the pathogenesis of PsA. Evidence suggests that a cross-talk exists between bone marrow inflammation and enthesitis, and that intestinal inflammation may also play a role in bone marrow involvement in axPsA. Subclinical intestinal inflammation is associated with increased joint disease activity and bone marrow edema in the joints [26,27,28,29]. Conversely, patients with chronic inflammatory bowel diseases (IBDs) often present with articular manifestations that fall within the spectrum of SpA [30].

At the core of the inflammatory process is the dysregulation of the IL-23/IL-17 axis, though with some distinctions:

AxPsA: IL-23 stimulates entheseal T cells to produce IL-17, promoting enthesitis and aberrant bone remodeling. Studies in murine models have shown that IL-23 is crucial for entheseal inflammation and periosteal ossification [9,31].

AS: IL-17 production is partly independent of IL-23, suggesting a greater reliance on memory T cells [32,33].

This difference may explain the ineffectiveness of IL-23 inhibitors in AS and their potential therapeutic role in axPsA. Although the IL-23/IL-17 axis is involved in the pathogenesis of both conditions, therapeutic responses differ—indeed, IL-23 inhibitors are not considered effective in AS, although they may be beneficial in axPsA [12,13].

### 1.5. Treatment

As mentioned, although axPsA and AS share many common features, they are characterized by clear clinical, radiographic, and pathogenetic differences. As a result, therapeutic responses also diverge significantly, requiring personalized treatment strategies [34]. The treatment of axPsA has been primarily based on therapeutic experience gained from peripheral PsA and AS. Currently, the GRAPPA [35,36] and EULAR guidelines do not recommend treating axPsA with IL-23 inhibitors, instead favoring the use of anti-TNFα agents, anti-IL-17 agents, or JAK inhibitors [37,38]. These recommendations stem from the lack of efficacy shown by therapies directly targeting IL-23, as demonstrated by clinical trials [39,40] for the treatment of AS, which—as previously highlighted by the studies of McGonagle and recent data—should be considered a distinct disease with different characteristics [13,14].

Post hoc analyses of the DISCOVER-1 and DISCOVER-2 trials suggest the potential benefit of IL-23 inhibitors, such as the biologic drug Guselkumab, in axPsA [41]. Currently, data remain limited, underscoring the importance of further research and clinical studies to fully explore the therapeutic potential of new biologic treatments across different inflammatory diseases, considering the diversity among the phenotypes included under the umbrella term “spondyloarthritis” (SpA).

Evidence suggests that disease involving the soft tissues of the axial enthesis distinguishes axial PsA from AS. This objective clinical distinction, along with other differences already described, highlights how it may be inappropriate to infer the ineffectiveness of IL-23 inhibition in axPsA based solely on its lack of efficacy in AS. In axPsA, a lower degree of cytokine neutralization might be sufficient to achieve a therapeutic effect in soft-tissue enthesis disease, as opposed to the more bone-driven pathology typical of AS. Ongoing international collaborations—such as GRAPPA and the Assessment of SpondyloArthritis International Society (ASAS)—are working to better understand axial disease in PsA through the AXIS study (axial involvement in psoriatic arthritis) [42], which aims to develop a universal definition and diagnostic criteria to enhance the understanding and management of axPsA. This is essential for more accurate disease classification and for developing more effective therapeutic strategies.

The efficacy of Guselkumab is also suggested by thorough analyses of DISCOVER-1 and DISCOVER-2 [43,44]. This provides the starting hypothesis for the present 12-month, real-life, single-center study conducted on PsA patients followed at the rheumatology unit of the “Gaspare Rodolico—San Marco” University Hospital in Catania.

Indeed, the aim of the study is to demonstrate, through real-life data, the efficacy of treatment with Guselkumab, as documented by the analysis of clinimetric parameters in 99 patients and by MRI evaluation in those identified as having axPsA. The primary endpoint of the study is to demonstrate a statistically significant improvement in clinimetric scores across the entire group treated with Guselkumab for 12 months. The secondary endpoint focuses on the statistically significant improvements observed in MRI scores among patients with clinical signs of axPsA.

## 2. Materials and Methods

This is a single-center, 12-month, longitudinal, single-arm, prospective observational cohort study conducted in a real-life setting. The cohort consisted of 99 patients with PsA followed at the outpatient clinics of the rheumatology unit of the AOUP Gaspare Rodolico San Marco, Catania, Italy of whom 58 presented with axial involvement (axPsA).

Informed consent was obtained from all participants prior to their inclusion in the study. The study was conducted in accordance with the principles of the Declaration of Helsinki.

All PsA patients met the CASPAR classification criteria for psoriatic arthritis.

Patients were treated with Guselkumab 100 mg, administered as a single subcutaneous injection at baseline (t_0_), followed by another injection after 4 weeks, and subsequently one subcutaneous injection every 8 weeks (as per the product label). The clinimetric indices used to evaluate the disease activity were mBASDAI, ASDAS, SJC, TJC, LEI, HAQ, PGA, VAS PAIN, and DAPSA.

Axial involvement was defined by the presence of inflammatory back pain fulfilling the ASAS criteria and confirmed by sacroiliac joint MRI demonstrating active bone marrow edema consistent with inflammatory sacroiliitis, as assessed using the SPARCC scoring method.

MRI of the SI joints was performed using a 3T MRI scanner (Siemens Magnetom Vida, Siemens Healthineers, Forchheim, Germany) with appropriate 16-channel surface coils. The sequence protocols were as follows: coronal (along the long axis of the sacral bone) T1-weighted (T1W) spin-echo (SE) (time to repeat/echo time [TR/TE], 362/9 ms; matrix, 256 166; slice thickness, 3 mm; field of view [FOV] 250; flip angle [FA], 150), and coronal fat-saturated (FS) T2-weighted (T2W) SE (TR/TE, 2460/62 ms; matrix, 256 166; slice thickness, 3 mm; FOV, 250; FA 150).

All MRIs were independently assessed by two musculoskeletal radiologists with over 10 years of experience, both blinded to the patients’ clinical data. Although no formal inter- or intra-reader reliability analysis (e.g., ICC) was performed, the use of dual independent evaluations aimed to enhance scoring consistency.

The SPARCC SI joint inflammation (SIS) and structural (SSS) scores were used in the evaluation of the SIJs.

The SPARCC SIS scoring system requires coronal T1W and short tau inversion recovery (STIR or T2FS) imaging of the SIJs. Each SIJ was divided into four quadrants (upper iliac, lower iliac, upper sacral, and lower sacral) and scored dichotomously for the presence of bone edema in six consecutive coronal slices, where 1 denotes an increased signal and 0 a normal signal, making a maximum total score of 48. In addition, the depth (≥1 cm = 1 point) and intensity (signal from presacral blood vessels = 1 point) of bone edema were separately scored for each slice, yielding a maximum score of 4 per slice. Increased signaling within the ligamentous portion of the SIJ was not scored. The total score of SIJ SIS ranged from 0 to 72.4. A score ≥2 for SIJ bone marrow edema was considered positive MRI evidence of active inflammation.

The SPARCC SSS was used to assess the structural lesions in each SIJ quadrant (fat metaplasia, erosion) and each half (backfill, ankylosis) in five slices of T1W images. Erosions and fat metaplasia were separately scored from 0 to 8 per slice (each maximum score 40), while backfill and ankylosis were separately scored from 0 to 4 per slice (each maximum score 20). The maximum score was 120 [45]. The follow-up examinations carried out one year later have not yet allowed us to assess the structural lesions.

Patients in the group with signs of axial involvement underwent MRI at baseline (t_0_), after 6 months (t_6_), and after 12 months of therapy (t_12_), assessing changes in MRI images following treatment with Guselkumab.

Clinimetric assessments were conducted at both 6 months and 12 months to determine whether patients exhibited statistically significant improvements (reductions in scores).

First, the Friedman test was used to assess whether the overall changes from baseline to 12 months were statistically significant. Given the significance of these results, further analyses were conducted to evaluate improvements between successive follow-up time points using the Wilcoxon signed-rank test.

The retention rate (RR) was calculated for the entire patient cohort and across subgroups defined by individual characteristics. Given the dataset structure, with only two discrete follow-up time points (6 months and 12 months) following baseline assessment, and the presence of censored patients, the life table method was employed to estimate the retention probability for different groups at these two follow-up time points.

To assess whether RR differed among patient subgroups, the log-rank test was performed. The only statistically significant differences were observed between patients with and without fibromyalgia, both across the entire follow-up period (*p* < 0.0001) and at each individual time point (*p* = 0.02 at 6 months, *p* < 0.0001 at 12 months).

Given the exploratory nature of the study and the limited number of pairwise comparisons, multiplicity correction procedures were not applied.

MRI assessment data in patients with axPsA were collected at baseline (t_0_), after 6 months (t_6_), and after 12 months (t_12_) of therapy. The data were analyzed using the Friedman test, followed by pairwise comparisons of scores between different follow-up time points using the Wilcoxon signed-rank test.

## 3. Results

### 3.1. Patient Characteristics

The study included a total of 99 Caucasian patients diagnosed with PsA, of whom 58 showed clinical signs of axial involvement (axPsA). The majority were women (72.3%). The mean age was 52.2 years (SD = ±12.1), with similar ranges between males (52 ± 13) and females (51.9 ± 12.1). 67% of patients were aged over 50. A total of 54 patients had at least one major comorbidity: 27.3% had cardiovascular diseases, 5% had obesity, and 34.2% had fibromyalgia. The mean BMI of the cohort was 28.3 (± 5.1), with slightly higher values in males (28.7 ± 4.3) than in females (28.1 ± 5.4). These averages place the population at the threshold between overweight and obesity—an important clinical consideration, as obesity is a known factor that can negatively impact the response to biologic treatments [46]. Among the 99 patients seen in the outpatient clinic, 93% reported peripheral joint pain, 7% had isolated axial pain, and 51.5% reported both peripheral and axial joint pain. Only 6.1% of patients were biologic-naïve at the time of study inclusion, with a predominance of female subjects. The remaining patients were enrolled due to the need for a biologic switch following prior therapeutic failure. While 15.1% of patients reported a family history of cutaneous psoriasis (PsO), with no significant sex differences, 27.3% reported a family history of psoriatic arthritis (PsA), with a higher prevalence among females (34.7%) than among males (7%). Finally, the study group reported a smoking habit in 24% of cases and presented with various comorbidities, including metabolic syndrome, thyroid disorders, cardiovascular issues such as hypertension and hypercholesterolemia, and, in some cases, associated anxiety/depressive syndrome and fibromyalgia. Table 1 and Table 2 summarize patients’ characteristics, disease features at baseline (t_0_) and comorbidities.

### 3.2. Clinimetric Evaluation

The results, presented in Table 3 below, indicate that all clinimetric measures showed highly significant improvements across the entire follow-up period and in pairwise comparisons between successive time points, except for ASDAS scores at 6 and 12 months, where no significant difference was observed. This suggests that the significant reduction in ASDAS scores occurred entirely within the first 6 months, with no further significant change between 6 and 12 months. Treatment success was defined as the achievement of statistically significant and consistent improvement in validated disease activity scores (DAPSA, ASDAS, and mBASDAI) and SPARCC MRI inflammation scores, reflecting both clinical and imaging responses.

Almost all 95% confidence intervals (CIs) between t_0_ and t_6_ or between t_0_ and t_12_ do not include zero, indicating a significant improvement with therapy. Some differences between t_6_ and t_12_ show CIs that approach or include zero, suggesting that the additional improvement after six months may be less pronounced or borderline. The combination of *p*-values and 95% CIs confirms a substantial clinical improvement during the first six months, with either consolidation of therapeutic benefits or slight further improvement by 12 months, depending on the parameter.

Table 4 essentially confirms the previously reported data, namely that the therapy led to a significant improvement in all clinical parameters between baseline and 6 months, with further benefit observed at 12 months. Additionally, analysis of IQR, P10, and P90 values shows a reduction, indicating that patients with more severe forms also benefited from the therapy.

**Figure 1:**
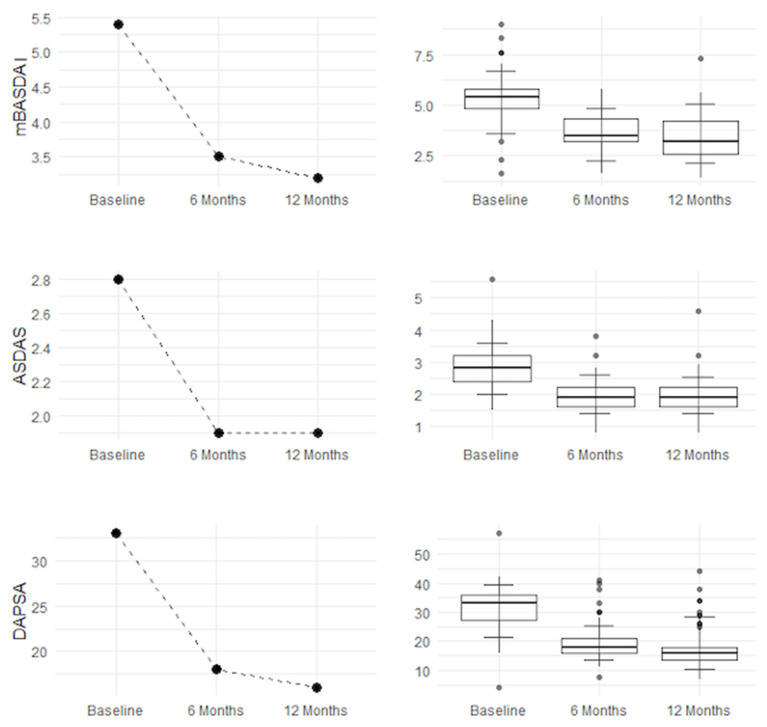
mBASDAI, ASDAS, and DAPSA improvements.

**Figure 2:**
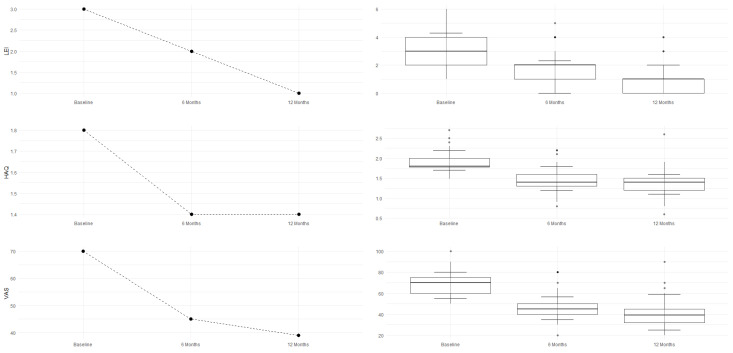
LEI, HAQ, and VAS PAIN improvements.

Improvement is evident both in disease activity parameters (mBASDAI, ASDAS, and DAPSA) and in clinical symptoms (VAS PAIN, LEI, and HAQ). Pain (VAS PAIN) and quality of life (HAQ) show sustained improvement up to 12 months, confirming the long-term efficacy of the treatment, as shown in Figure 1 and Figure 2.

### 3.3. Radiological Evaluation

The MRI assessment results for four parameters regarding SPARCC were right score, left score, L + R score, and intensity, analyzing the changes from baseline (t_0_) to the follow-ups at 6 months (t_6_) and 12 months (t_12_). According to the Friedman test, shown in Table 5, the *p*-values for left score, L + R score, and intensity are ≤0.0024, indicating a statistically significant difference across time points. The right score has a *p*-value of 0.0175, which is less significant than for the other parameters but still below the threshold of *p* < 0.05—indicating a specific and effective improvement, as detected by imaging. The intensity of inflammation (intensity) continues to decrease up to 12 months, suggesting a possible prolonged therapeutic effect. When examining the confidence intervals (CIs), a statistically significant improvement is observed during the first 6 months for all evaluated scores, followed by a consolidation of the results by 12 months.

**Figure 3:**
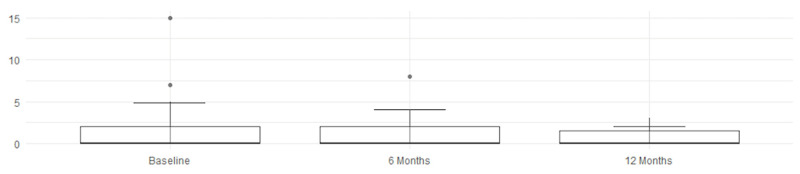
Right score improvement.

**Figure 4:**
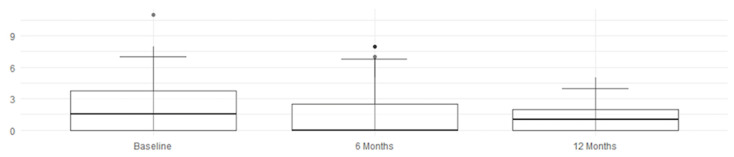
Left score improvement.

**Figure 5:**
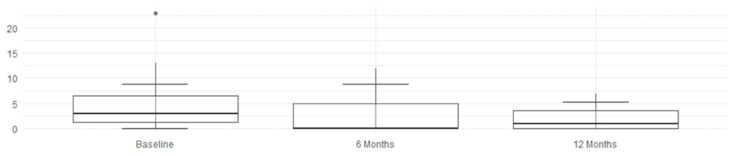
L + R score improvement.

**Figure 6:**
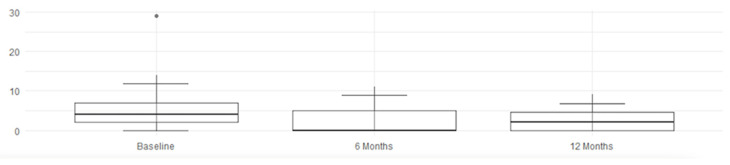
Intensity score improvement.

An overall and immediate view—highlighting the statistical significance of the therapeutic benefits—is clearly shown by these graphs and histograms illustrating the decrease in MRI scores in Figure 3, Figure 4, Figure 5 and Figure 6.

Finally, Table 6 reports the SPARCC values of right score, left core, L + R score, and intensity, including their medians, interquartile ranges (IQRs), and 10th (P10) and 90th (P90) percentiles, which confirm the trend of improvement. The reduction in median and P90 values suggests a decrease in the severity of the MRI-detected alterations over time. A lower IQR, indicating reduced data dispersion over time, reflects a more consistent response to therapy; nearly all values show clear improvement, while the L +R score exhibits slight fluctuations.

### 3.4. Assessment of Guselkumab Retention Rate

**Figure 7:**
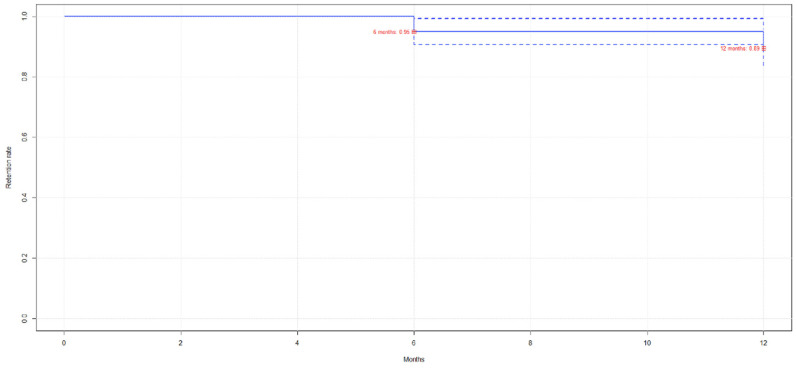
Guselkumab retention rate (Kaplan–Meier)**.** Red squares indicate the cumulative retention rate measured at 6-month and 12-month follow-up points.

As shown in Table 7 and Figure 7, the drug retention rate remains nearly constant over time, although it slightly decreases at 12 months in some patient groups.

### 3.5. Assessment of Retention Rate in Patients with Fibromyalgia Comorbidity

**Figure 8:**
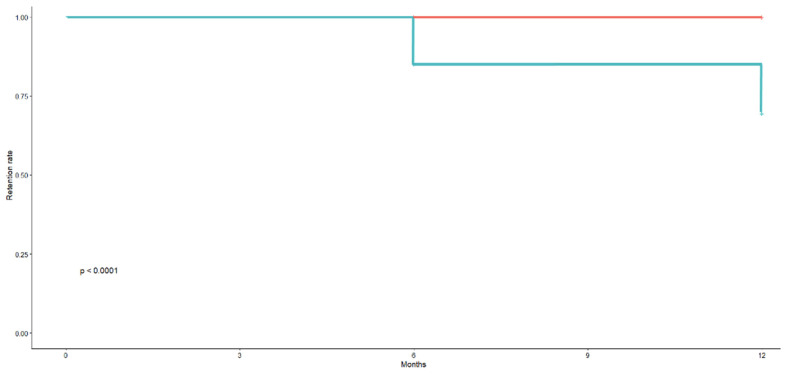
Kaplan–Meier curve for the retention rate of Guselkumab in PsA patients in relation to fibromyalgia. The blue line represents patients with fibromyalgia, while the red line represents those without.

As shown in Figure 8, we stratified the presence or absence of fibromyalgia.

Red line → Patients without fibromyalgia maintain a high retention rate up to 12 months.

Blue line → Patients with fibromyalgia show a significant drop in retention at around 6 months, with a further decline by 12 months. The graph indicates a highly significant difference between the two groups, suggesting that fibromyalgia negatively impacts treatment retention. Patients with fibromyalgia are more likely to discontinue therapy, possibly due to a perceived lower clinical response, increased pain sensitivity, side effects, or reduced tolerability.

## 4. Discussion

These results were collected in real life from 99 patients at baseline, 87 patients at 6 months, and 64 patients at 12 months. Some patients were lost to follow-up: eight discontinued due to lack of efficacy, one experienced an adverse reaction to the drug, and another switched to Apremilast due to worsening renal function in a diabetic patient with chronic kidney disease. The data collected show significant decreases in all clinical and quality-of-life parameters at both 6 and 12 months of therapy in PsA patients. This suggests that treatment with Guselkumab was effective not only in reducing disease activity and reported pain but also in improving physical function and quality of life.

All evaluated clinimetric indices showed a statistically significant improvement between baseline, 6 months, and 12 months, with a notable reduction in inflammation and signs of remission in many patients. This prospective study on PsA patients over a 12-month period confirms that therapy with Guselkumab is effective in the routine clinical management of these patients, helping them reach individual minimal disease activity (MDA) criteria—essential for reducing disease burden and improving quality of life.

MRI evaluation in patients with axial involvement shows, contrary to previous assumptions, that Guselkumab is statistically effective in improving bone marrow edema in the sacroiliac joints and reducing psoriatic inflammation in the axial skeleton, as demonstrated by the reduction in MRI scores at follow-up. These results support the efficacy of Guselkumab in treating not only peripheral PsA but also axial PsA. The observed reduction in clinimetric indices and MRI parameters from our outpatient setting aligns closely with data from the DISCOVER-1 and DISCOVER-2 clinical trials [43,44].

**Figure 9:**
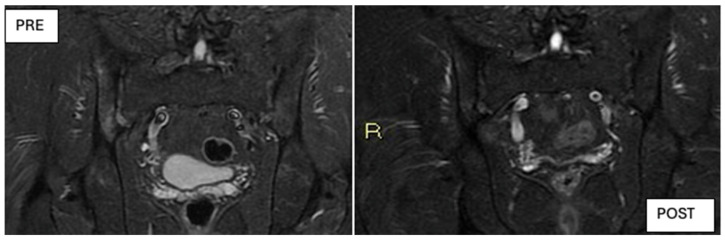
MRI images at baseline (t_0_, **left**) and after 6 months of treatment with Guselkumab (t_6_, **right**) showing a clear reduction in bone marrow edema and inflammatory lesions.

Although DISCOVER-1 and DISCOVER-2 were not originally designed to specifically evaluate axial PsA, a subsequent post hoc analysis assessed outcomes in a subgroup of patients with axial symptoms and MRI-confirmed sacroiliitis. That study reported improvements in ASDAS, BASDAI, and SPARCC MRI scores, which are consistent with the MRI findings shown in Figure 9, where notable improvements are observed following treatment with Guselkumab.

The consistency between those findings and our real-world data supports the potential role of Guselkumab in axial disease, even outside the context of formal RCT endpoints.

Treatment success was assessed through reductions in clinimetric scores, with long-term efficacy demonstrated by sustained reductions in both axial symptoms and overall disease activity. The clinimetric data are consistent with the post hoc analysis of both DISCOVER-1 and DISCOVER-2. Indeed, pooled post hoc analyses of PsA patients (pts) with investigator-confirmed sacroiliitis (prior imaging or radiograph at screening) showed that GUS-treated pts had significantly greater improvements in axial-related symptoms (evaluated by BASDAI, modified BASDAI, and ASDAS-CRP) vs. placebo at week (W) 24, with improvements maintained through W52. Moreover, greater improvements in axial-related symptoms were confirmed for GUS-treated patients, regardless of HLA-B27 status [43,44]. Moreover, they demonstrated not only reduced clinical symptoms but also led to significant reductions in inflammation, as assessed by MRI.

Indeed, in this cohort, radiologic improvement was consistently associated with clinical improvements in ASDAS and mBASDAI scores. No clear discordant cases were observed, suggesting a good correlation between imaging findings and symptom reduction. This highlights the potential of Guselkumab to treat not only symptoms but also the underlying inflammatory processes in PsA. The analysis of the drug’s retention rate shows that it remains high over time, with only a slight decrease between 6 and 12 months.

A retention rate greater than 90% at both 6 and 12 months is generally considered a positive outcome in long-term treatments, suggesting that the drug is well tolerated and effective for most patients. Only patients with fibromyalgia comorbidity showed a slight decline in RR over time, supporting the need for personalized treatment strategies in this subgroup. Indeed, in the small subset of patients with comorbid fibromyalgia who discontinued Guselkumab, discontinuation appeared to be driven by a subjective perception of limited efficacy rather than safety issues. The distinction between fibromyalgia-related symptoms and PsA disease activity is clinically challenging, as the two conditions may overlap in domains such as pain and fatigue. This diagnostic ambiguity is a recognized limitation of current clinimetric instruments and remains an ongoing topic of debate in the literature, particularly in the context of PsA’s heterogeneous clinical presentation.

The results support the initial hypothesis that significant improvements can be observed in patients with PsA and axial involvement before and after treatment with Guselkumab.

Indeed, while the inflammatory process is driven by dysregulation of the IL-23/IL-17 axis in both conditions, IL-23 plays a central role in entheseal inflammation and periosteal ossification in axPsA, unlike in AS, where IL-17 production is partly IL-23-independent and more reliant on memory T cells. This explains the differing response to Guselkumab between the two diseases and why IL-23 inhibitors are not considered effective in AS but may be beneficial in axPsA [13].

We acknowledge that the single-center design of our study, along with the relatively small sample size of the axPsA subgroup (n = 58), may have introduced selection bias, potentially affecting the representativeness and external validity of our findings. While our results are encouraging and align with previous evidence in the literature, to strengthen and validate these observations, future studies will be essential. Moreover, while a formal control group was not included, the real-world nature of the study still provides meaningful information on the effectiveness of Guselkumab in a broad PsA population. Although some confounding factors, such as concomitant therapies and disease duration, cannot be completely ruled out, the consistent improvements seen across multiple clinical and imaging outcomes support the reliability of the results. In addition, future studies are warranted to directly compare the efficacy of IL-23 inhibitors, such as Guselkumab, in patients with axPsA and those with AS. Such research would be essential to clarify the extent to which pathogenetic differences between these two conditions influence treatment responses, ultimately guiding more tailored therapeutic strategies for each disease entity. Furthermore, although biomarkers such as IL-23 and IL-17 were not assessed in the initial phase of the study, CRP levels were systematically collected. An extension of this cohort is currently being planned to include prospective biomarker analysis and correlation with clinical and imaging responses, aiming to better understand treatment mechanisms and optimize patient stratification. Although no formal stratified analysis was performed, baseline characteristics—including HLA-B27 status, joint involvement pattern, prior biologic or csDMARD use, and comorbidities—were systematically collected and reported in Table 1. These variables were considered during the interpretation of response trends. Future subgroup analyses are planned to further explore the potential impact of these factors. Finally, an extended follow-up is ongoing to monitor long-term clinical and radiologic outcomes beyond 12 months, including structural MRI lesions and flare rates. Based on the sustained reduction in inflammation observed during the first year, it is expected that clinical improvement could be maintained, and that radiologic progression might remain limited with continued Guselkumab therapy.

Moreover, although BMI and comorbidities, such as metabolic syndrome, were collected and reported (Table 1), we did not perform a dedicated analysis at this time to assess their potential influence on treatment response. As these factors are increasingly recognized as possible modifiers of disease activity and therapeutic outcomes in PsA, future studies will be needed to further explore their role, particularly in real-world settings.

Finally, the single-center design and the predominance of female patients may limit the external validity of the study, as this distribution may not fully reflect the broader PsA population. However, the inclusion of 99 patients provides a meaningful dataset for a real-world, monocentric setting. These aspects should be taken into account when interpreting the findings.

## 5. Conclusions

This is the first real-world, 12-month prospective study on 99 outpatients, and it demonstrates that Guselkumab is effective and safe in the routine clinical management of psoriatic arthritis (PsA), whether peripheral or axial. The reduction in disease severity associated with this treatment is closely linked to improvements in quality of life within the same patient group.

The analysis of the data collected in this RWE study provides statistically significant evidence of Guselkumab’s efficacy in reducing key disease activity parameters in PsA, as measured by clinimetric tests and MRI scoring. These results confirm the findings of previous DISCOVER-1 and DISCOVER-2 trials and other real-world studies of shorter duration [47].

MRI-based measurements show improvements in disease burden, inflammation intensity, bone marrow edema, and lesion distribution areas, providing strong evidence for the effectiveness and safety of Guselkumab, even in patients with axPsA.

Moreover, these data support the use of Guselkumab as an effective and well-tolerated therapeutic option, with a positive impact on both axial and peripheral manifestations of psoriatic arthritis.

Further follow-up may help determine whether this trend of improvement is sustained over the longer term while awaiting the results of the Phase 4 STAR study, which will evaluate the efficacy and safety of Guselkumab in bio-naïve patients with active axPsA [48].

## Figures and Tables

**Table 1:** Patients’ characteristics and disease features at baseline (t_0_).

PsA Patients	All Patients	Males	Females
Total number (%)	99 (100%)	27 (27.7%)	72 (72.3%)
Gender, female, n (%)	72 (72.3%)	0 (0%)	72 (100%)
Gender, male, n (%)	27 (27.7%)	27 (100%)	0 (0%)
Age, years ± SD	52.2 ± 12.1	52.1 ± 13	51.9 ± 12.1
BMI, value ± SD	28.3 ± 5.1	28.7 ± 4.3	28.1 ± 5.4
Disease Features, baseline			
Family history of PsO, n. (%)	15 (15.1%)	4 (14.8%)	11 (15.3%)
Family history of PsA, n. (%)	27 (27.3%)	2 (0.7%)	25 (34.7%)
DAPSA at baseline, mean ± SD	31.2 ± 7.5	31.8 ± 6.1	30.9 ± 8
ASDAS-CRP at baseline, mean ± SD	2.83 ± 0.7	2.7 ± 1	3.2 ± 1
mBASDAI at baseline, mean ± SD	5.3 ± 1.3	4.7 ± 1.7	5.4 ± 1.3
Bio-naïve, n. (%)	6 (6.1%)	1 (0.4%)	5 (0.7%)
Prev. sDmards	45 (45.4%)	17(17.2%)	28 (28.3%)

**Table 2:** Patients’ comorbidities.

Comorbidities, Baseline	Patients
Hypertension	24 (24.2%)
Cardiovascular diseases	3 (3%)
Respiratory diseases	1 (1%)
Psoriasis	19 (19.2%)
Fibromyalgia	34 (34.2%)
Diabetes	9 (0.9%)
Methabolic syndrome	12 (12.1%)
Thyroid disease	21 (21.2%)
B and C Hepatitis	6 (6.1%)
Quantiferon positivity	7 (7.1%)
Solid tumors	3 (3%)
Blood tumors	1 (1%)
Anxiety/Depression	9 (9.1%)
Kidney failure	2 (2%)
Osteopenia/Osteoporosis	6 (6.1%)
IBD	1 (1%)

**Table 3:** Clinimetric evaluation.

Clinimetric Evaluations	Friedman Statistic		Median								
	Estimate	*p*-Value	t_0_ vs. t_6_	*p*-Value	t_0_ vs. t_12_	*p*-Value	t_6_ vs. t_12_	*p*-Value	CI(95%) t_0_ vs. t_6_	CI(95%) t_0_ vs. t_12_	CI(95%) t_6_ vs. t_12_
mBASDAI	53.47	0.0000	1.8	0.0000	2.1	0.0000	0.4	0.0297	(1.4, 2.2)	(1.5, 2.5)	(−0.3, 0.6)
ASDAS	42.82	0.0000	0.8	0.0000	2.1	0.0000	0.1	0.4606	(0.6, 1.0)	(0.7, 1.1)	(−0.2, 0.3)
SJC	75.72	0.0000	3.0	0.0000	4.0	0.0000	0.0	0.001	(2.0, 4.0)	(4.0, 4.0)	(0.0, 2.0)
TJC	75.68	0.0000	7.0	0.0000	8.0	0.0000	2.0	0.0416	(5.0, 8.0)	(7.0, 11.0)	(2.0, 3.0)
LEI	99.34	0.0000	2.0	0.0000	2.0	0.0000	0.0	0.004	(1.0, 2.0)	(2.0, 2.0)	(0.0, 1.0)
HAQ	92.99	0.0000	0.4	0.0000	0.5	0.0000	0.1	0.0036	(0.3, 0.5)	(0.4, 0.6)	(0.0, 0.2)
PGA	81.23	0.0000	2.5	0.0000	30.0	0.0000	5.0	0.0487	(20.0, 25.0)	(20.0, 30.0)	(0.0, 10.0)
VAS PAIN	83.12	0.0000	2.0	0.0000	30.0	0.0000	5.0	0.0014	(20.0, 29.5)	(25.0, 35.0)	(1.0, 10.0)
DAPSA	90.30	0.0000	14.0	0.0000	17.0	0.0000	2.0	0.0013	(14.0, 16.5)	(16.0, 19.0)	(1.0, 3.0)

**Table 4:** Clinimetric outcomes over 12 months of Guselkumab treatment.

Variable	Follow-Up (months)	Median	IQR	P_10_	P_90_
mBASDAI	Baseline	5.40	1.00	3.60	6.70
	6	3.50	1.10	2.26	4.80
	12	3.20	1.60	2.15	5.03
ASDAS	Baseline	2.80	0.80	2.00	3.60
	6	1.90	0.60	1.40	2.58
	12	1.90	0.60	1.38	2.53
LEI	Baseline	3.00	2.00	2.00	4.30
	6	2.00	1.00	0.00	2.30
	12	1.00	1.00	0.00	2.00
HAQ	Baseline	1.80	0.22	1.70	2.20
	6	1.40	0.30	1.20	1.80
	12	1.40	0.30	1.10	1.60
VAS	Baseline	7.00	15.00	55.00	80.00
	6	45.00	10.00	35.00	56.50
	12	39.00	13.00	25.00	59.00
DAPSA	Baseline	33.00	8.50	21.40	39.30
	6	18.00	5.00	13.70	25.30
	12	16.00	4.50	10.20	28.40

**Table 5:** MRI SPARCC SIS (sacroiliac joint inflammation score) evaluations.

MRI Evaluations	Friedman Statistic		Median								
	Estimate	*p*-Value	t_0_ vs. t_6_	*p*-Value	t_0_ vs. t_12_	*p*-Value	t_6_ vs. t_12_	*p*-Value	CI(95%) t_0_ vs. t_6_	CI(95%) t_0_ vs. t_12_	CI(95%) t_6_ vs. t_12_
Right Score	8.09	0.0175	0	0.1390	0	0.0310	0	0.4098	(0, 2.0)	(−0.5, 2.0)	(−1.0, 1.0)
Left Score	12.05	0.0024	0	0.0842	2	0.0052	0	0.5034	(0, 3.0)	(−1.0, 2.0)	(−2.0, 1.0)
L + R Score	16.68	0.0002	1	0.0207	2	0.0015	1	0.2148	(0, 4.0)	(0, 3.5)	(−3.0, 2.5)
Intensity	17.43	0.0002	1.5	0.0023	3	0.0015	1	0.4530	(0.5, 6.0)	(0, 5.0)	(−4.0, 3.0)

**Table 6:** SPARCC SIS median follow-up.

Variable	Follow-Up (months)	Median	IQR	P_10_	P_90_
Right Score	Baseline	0.00	2.00	0.00	4.90
	6	0.00	2.00	0.00	4.00
	12	0.00	1.50	0.00	2.00
Left Score	Baseline	1.50	3.75	0.00	7.00
	6	0.00	2.50	0.00	6.80
	12	1.00	2.00	0.00	4.00
L + R Score	Baseline	3.00	5.25	0.00	8.90
	6	0.00	5.00	0.00	8.90
	12	1.00	3.50	0.00	5.20
Intensity	Baseline	4.00	5.00	0.00	11.90
	6	0.00	5.00	0.00	8.90
	12	2.00	4.50	0.00	6.80

**Table 7:** Guselkumab retention rate.

		Time	No. Risk	No. Event	Retention Rate	Cum. Ret. Rate
Overall	Groups	6	99	5	0.947	0.947
		12	87	5	0.940	0.890
Age	Over 50	6	66	3	0.953	0.953
		12	66	4	0.897	0.855
	Under 50	6	33	2	0.938	0.938
		12	33	1	0.947	0.888
Sex	Female	6	74	4	0.944	0.944
		12	74	3	0.931	0.879
	Male	6	25	1	0.959	0.959
		12	25	2	0.862	0.827

## Data Availability

The data that support the findings of this study are available from the corresponding author upon reasonable request.

## Abbreviations

The following abbreviations are used in this manuscript:

mBASDAI	Modified Bath Ankylosing Spondylitis Disease Activity Index
ASDAS	Ankylosing Spondylitis Disease Activity Score
SJC	Swollen Joint Count
TJC	Tender Joint Count
LEI	Leeds Enthesitis Index
HAQ	Health Assessment Questionnaire
PGA	Patient Global Assessment
VAS PAIN	Visual Analogue Scale for Pain
DAPSA	Disease Activity Index for Psoriatic Arthritis

## References

[1] Coates L.C., Helliwell P.S. (2017). Psoriatic arthritis: State of the art review. Clin. Med..

[2] Cho H.H., Kim B.S. (2013). Psoriatic arthritis: A literature review. J. Lifestyle Med..

[3] López-Ferrer A., Laiz A., Puig L. (2022). Psoriatic arthritis. Med. Clin..

[4] Wright V. (1978). Seronegative polyarthritis: A unified concept. Arthritis Rheum..

[5] Poddubnyy D., Jadon D.R., Bosch F.V.D., Mease P.J., Gladman D.D. (2021). Axial involvement in psoriatic arthritis: An update for rheumatologists. Semin. Arthritis Rheum..

[6] Chandran V. (2019). Psoriatic spondylitis or ankylosing spondylitis with psoriasis: Same or different?. Curr. Opin. Rheumatol..

[7] Bagel J., Schwartzman S. (2018). Enthesitis and Dactylitis in Psoriatic Disease: A Guide for Dermatologists. Am. J. Clin. Dermatol..

[8] Yao Y., Richman L., Morehouse C., de los Reyes M., Higgs B.W., Boutrin A., White B., Coyle A., Krueger J., Kiener P.A. (2008). Type I interferon: Potential therapeutic target for psoriasis?. PLoS ONE.

[9] Mease P., van den Bosch F. (2021). IL-23 and axial disease: Do they come together?. Rheumatology.

[10] Fitch E., Harper E., Skorcheva I., Kurtz S.E., Blauvelt A. (2007). Pathophysiology of psoriasis: Recent advances on IL-23 and Th17 cytokines. Curr. Rheumatol. Rep..

[11] McGonagle D.G., McInnes I.B., Kirkham B.W., Sherlock J., Moots R. (2019). The role of IL-17A in axial spondyloarthritis and psoriatic arthritis: Recent advances and controversies. Ann. Rheum. Dis..

[12] McGonagle D., Watad A., Sharif K., Bridgewood C. (2021). Why Inhibition of IL-23 Lacked Efficacy in Ankylosing Spondylitis. Front. Immunol..

[13] McGonagle D., David P., Macleod T., Watad A. (2023). Predominant ligament-centric soft-tissue involvement differentiates axial psoriatic arthritis from ankylosing spondylitis. Nat. Rev. Rheumatol..

[14] Bridgewood C., Sharif K., Sherlock J., Watad A., McGonagle D. (2020). Interleukin-23 pathway at the enthesis: The emerging story of enthesitis in spondyloarthropathy. Immunol0 Rev..

[15] Michelena X., Poddubnyy D., Marzo-Ortega H. (2020). Axial Psoriatic Arthritis: A Distinct Clinical Entity in Search of a Definition. Rheum. Dis. Clin. N. Am..

[16] Scotti L., Franchi M., Marchesoni A., Corrao G. (2018). Prevalence and incidence of psoriatic arthritis: A systematic review and meta-analysis. Semin. Arthritis Rheum..

[17] Huskisson E.C. (1974). Measurement of pain. Lancet.

[18] Helliwell P.S., Hickling P., Wright V. (1998). Do the radiological changes of classic ankylosing spondylitis differ from the changes found in the spondylitis associated with inflammatory bowel disease, psoriasis, and reactive arthritis?. Ann. Rheum. Dis..

[19] Helliwell P.S. (2020). Axial disease in psoriatic arthritis. Rheumatology.

[20] Garrett S., Jenkinson T., Kennedy L.G., Whitelock H., Gaisford P., Calin A. (1994). A new approach to defining disease status in ankylosing spondylitis: The Bath Ankylosing Spondylitis Disease Activity Index. J. Rheumatol..

[21] Jadon D.R., Sengupta R., Nightingale A., Lindsay M., Korendowych E., Robinson G., Jobling A., Shaddick G., Bi J., Winchester R. (2017). Axial Disease in Psoriatic Arthritis study: Defining the clinical and radiographic phenotype of psoriatic spondyloarthritis. Ann. Rheum. Dis..

[22] Eshed I., Bollow M., McGonagle D.G., Tan A.L., Althoff C.E., Asbach P., Hermann K.G. (2007). MRI of enthesitis of the appendicular skeleton in spondyloarthritis. Ann. Rheum. Dis..

[23] Hetland M.L., Ejbjerg B., Hørslev-Petersen K., Jacobsen S., Vestergaard A., Jurik A.G., Stengaard-Pedersen K., Junker P., Lottenburger T., Hansen I. (2009). MRI bone oedema is the strongest predictor of subsequent radiographic progression in early rheumatoid arthritis. Ann. Rheum. Dis..

[24] Celis R., Planell N., Fernández-Sueiro J.L., Sanmartí R., Ramírez J., González-Álvaro I., Pablos J.L., Cañete J.D. (2012). Synovial cytokine expression in psoriatic arthritis and associations with lymphoid neogenesis and clinical features. Arthritis Res. Ther..

[25] Lee E., Trepicchio W.L., Oestreicher J.L., Pittman D., Wang F., Chamian F., Dhodapkar M., Krueger J.G. (2004). Increased expression of interleukin 23 p19 and p40 in lesional skin of patients with psoriasis vulgaris. J. Exp. Med..

[26] Schett G., Rahman P., Ritchlin C., McInnes I.B., Elewaut D., Scher J.U. (2022). Psoriatic arthritis from a mechanistic perspective. Nat. Rev. Rheumatol..

[27] Tateiwa D., Yoshikawa H., Kaito T. (2019). Cartilage and Bone Destruction in Arthritis: Pathogenesis and Treatment Strategy: A Literature Review. Cells.

[28] Mauro D., Gandolfo S., Tirri E., Schett G., Maksymowych W.P., Ciccia F. (2023). The bone marrow side of axial spondyloarthritis. Nat. Rev. Rheumatol..

[29] Hedin C.R.H., Sonkoly E., Eberhardson M., Ståhle M. (2021). Inflammatory bowel disease and psoriasis: Modernizing the multidisciplinary approach. J. Intern. Med..

[30] Najarian D.J., Gottlieb A.B. (2003). Connections between psoriasis and Crohn’s disease. J. Am. Acad. Dermatol..

[31] Sherlock J.P., Joyce-Shaikh B., Turner S.P., Chao C.C., Sathe M., Grein J., Gorman D.M., Bowman E.P., McClanahan T.K., Yearley J.H. (2012). IL-23 induces spondyloarthropathy by acting on ROR-γt+ CD3+CD4-CD8- entheseal resident T cells. Nat. Med..

[32] Gracey E., Qaiyum Z., Almaghlouth I., Lawson D., Karki S., Avvaru N., Zhang Z., Yao Y., Ranganathan V., Baglaenko Y. (2016). IL-7 primes IL-17 in mucosal-associated invariant T (MAIT) cells, which contribute to the Th17-axis in ankylosing spondylitis. Ann. Rheum. Dis..

[33] Taylan A., Sari I., Kozaci D.L., Yuksel A., Bilge S., Yildiz Y., Sop G., Coker I., Gunay N., Akkoc N. (2012). Evaluation of the T helper 17 axis in ankylosing spondylitis. Rheumatol. Int..

[34] Yousif P., Nahra V., Khan M.A., Magrey M. (2024). Disease characteristics, pathogenesis, and treatment controversies of axial psoriatic arthritis. Jt. Bone Spine.

[35] Leung Y.Y., Korotaeva T.V., Candia L., Pedersen S.J., Molano W.B., Ruderman E.M., Bisoendial R., Perez-Alamino R., Olsder W., Möller B. (2023). Management of peripheral arthritis in patients with psoriatic arthritis: An updated literature review Informing the 2021 GRAPPA treatment recommendations. J. Rheumatol..

[36] Coates L.C., Corp N., van der Windt D.A., O’Sullivan D., Soriano E.R., Kavanaugh A. (2022). GRAPPA Treatment Recommendations: 2021 Update. J. Rheumatol..

[37] Gossec L., Coates L.C., de Wit M., Kerschbaumer A., Ferreira R.J.O., Aletaha D., Baraliakos X., Bertheussen H., Boehncke W.-H., Esbensen B.A. (2020). EULAR recommendations for the management of psoriatic arthritis with pharmacological therapies: 2019 update. Ann. Rheum. Dis..

[38] Gossec L., Baraliakos X., Kerschbaumer A., Ferreira R.J.O., Aletaha D., Bertheussen H., Boehncke W.-H., Esbensen B.A., McInnes I.B., McGonagle D. (2024). EULAR recommendations for the management of psoriatic arthritis with pharmacological therapies: 2023 update. Ann. Rheum. Dis..

[39] Deodhar A., Gensler L.S., Sieper J., Clark M., Calderon C., Wang Y., Zhou Y., Leu J.H., Campbell K., Sweet K. (2019). Three Multicenter, Randomized, Double-Blind, Placebo-Controlled Studies Evaluating the Efficacy and Safety of Ustekinumab in Axial Spondyloarthritis. Arthritis Rheumatol..

[40] Baeten D., Østergaard M., Wei J.C.-C., Sieper J., Järvinen P., Tam L.-S., Salvarani C., Kim T.-H., Solinger A., Datsenko Y. (2018). Risankizumab, an IL-23 inhibitor, for ankylosing spondylitis: Results of a randomised, double-blind, placebo-controlled, proof-of-concept, dose-finding phase 2 study. Ann. Rheum. Dis..

[41] McGonagle D., McInnes I.B., Deodhar A., Schett G., Shawi M., Kafka S., Karyekar C.S., Kollmeier A.P., Hsia E.C., Xu X.L. (2021). Resolution of enthesitis by guselkumab and relationships to disease burden: 1-year results of two phase 3 psoriatic arthritis studies. Rheumatology.

[42] Poddubnyy D., Baraliakos X., Van den Bosch F., Braun J., Coates L.C., Chandran V., Diekhoff T., van Gaalen F.A., Gensler L.S., Goel N. (2021). Axial Involvement in Psoriatic Arthritis cohort (AXIS): The protocol of a joint project of the Assessment of SpondyloArthritis international Society (ASAS) and the Group for Research and Assessment of Psoriasis and Psoriatic Arthritis (GRAPPA). Ther. Adv. Musculoskelet. Dis..

[43] Deodhar A., Helliwell P.S., Boehncke W.-H., Kollmeier A.P., Hsia E.C., Subramanian R.A., Xu X.L., Sheng S., Agarwal P., Zhou B. (2020). Guselkumab in patients with active psoriatic arthritis who were biologic-naive or had previously received TNFα inhibitor treatment (DISCOVER-1): A double-blind, randomised, placebo-controlled phase 3 trial. Lancet.

[44] Mease P.J., Rahman P., Gottlieb A.B., Kollmeier A.P., Hsia E.C., Xu X.L., Sheng S., Agarwal P., Zhou B., Zhuang Y. (2020). Guselkumab in biologic-naive patients with active psoriatic arthritis (DISCOVER-2): A double-blind, randomised, placebo-controlled phase 3 trial. Lancet.

[45] Gezer H.H., Duruöz M.T. (2022). The value of SPARCC sacroiliac MRI scoring in axial psoriatic arthritis and its association with other disease parameters. Int. J. Rheum. Dis..

[46] Aljohani R. (2022). Metabolic Syndrome and Its Components in Psoriatic Arthritis. Open Access Rheumatol..

[47] Ruscitti P., Pantano I., Cataldi G., Gentile M., Arrigoni F., Riccio L., Marrone S., Mauro D., Ursini F., Esposito M. (2024). Short-term effectiveness of guselkumab in psoriatic arthritis patients and axial involvement: Results from a real-life multicentre cohort. Rheumatology.

[48] https://clinicaltrials.gov/study/NCT04929210?cond=Psoriatic%20Arthritis&term=star&rank=1#contacts-and-locations.

